# A Novel Variant c.97C>T of the Growth Hormone Releasing Hormone Receptor Gene Causes Isolated Growth Hormone Deficiency Type Ib

**DOI:** 10.4274/jcrpe.5188

**Published:** 2018-07-31

**Authors:** Assimina Galli-Tsinopoulou, Eleni P. Kotanidou, Aggeliki N. Kleisarchaki, Rivka Kauli, Zvi Laron

**Affiliations:** 1 Aristotle University of Thessaloniki Faculty of Health Sciences, School of Medicine, Papageorgiou General Hospital, 4^th^ Department of Pediatrics, Thessaloniki, Greece; 2 Tel Aviv University Sackler Faculty of Medicine, Schneider Children’s Medical Center of Israel, Clinic of Endocrinology and Diabetes Research, Tel Aviv, Israel

**Keywords:** Congenital isolated growth hormone deficiency, growth hormone releasing hormone receptor, failure to thrive, short stature

## Abstract

Congenital isolated growth hormone deficiency (IGHD) type 1b is an autosomal recessive genetic condition caused by mutations of growth hormone (GH)-1 or the growth hormone releasing hormone receptor (*GHRH-R*) genes. Affected subjects present with symptoms of growth hormone deficiency (GHD) with low but detectable levels of growth hormone (GH), short stature and responsiveness to GH therapy. We describe a 13-month old girl with severe growth failure who showed a low GH response to two GH provocation tests and a modest increase of insulin-like growth factor-1 (IGF-1) to an IGF-1 generation test. Whole exome sequencing revealed a novel homozygous variant of the *GHRH-R* gene (c.97C>T), leading to a premature stop codon. Administration of recombinant human GH improved linear growth. This is the first report of a c.97C>T mutation of the *GHRH-R* gene.

## What is already known on this topic?

Isolated growth hormone deficiency (IGHD) is a sporadic disease with insufficient or deficient production of growth hormone (GH). IGHD type 1b is caused by mutations in either the *GH-1* gene or the growth hormone releasing hormone receptor gene.

## What this study adds?

We report a previously undescribed genetic defect, the c.97C>T variant of the growth hormone releasing hormone receptor gene, which results in severe growth retardation, approaching growth arrest, in the homozygous state. The present case provides new data on genetic causes of isolated growth hormone deficiency type 1b and describes the phenotype of this novel mutation.

## Introduction

Isolated growth hormone deficiency (IGHD) is a sporadic disease with a prevalence ranging from 1:3480 to 1:10 000 live births (1). It is defined as an insufficient or deficient production of growth hormone (GH) by the pituitary gland. Its complex etiology involves a spectrum of hypothalamic defects, pituitary abnormalities or combined conditions, which can be structurally detected by brain imaging in only 26.8% of affected patients (2). 

Familial IGHD is classified into four distinct types with different clinical manifestation and inheritance patterns. The two most frequent types of IGHD are types 1a and 1b, characterized by an autosomal recessive trait; type 2 is transmitted as an autosomal dominant defect while type 3 appears with an X-linked inheritance pattern. Type 1a IGHD presents as an entire *GH-1* gene deletion with undetectable serum GH levels, extremely short stature and possible development of anti-GH antibodies after recombinant human GH administration (3,4). Type 1b IGHD presents with a milder phenotype, caused by mutations to either the *GH-1 *gene or to the GH releasing hormone receptor (*GHRH-R*) gene with low but detectable levels of serum GH, short stature and a positive response to GH therapy with immunologic tolerance (5). Type 2 IGHD patients may also present with low serum GH levels without development of anti-GH antibodies. IGHD-type 3 has been associated with occasional agammaglobulinemia (6).

The *GHRH-R * gene is located on the short arm of chromosome 7. A number of mutations within the specific locus of the *GHRH-R* gene have been reported in IGHD type 1b subjects, leading to loss of the receptor function and thus to growth failure. We present a novel mutation of the GHRH-R, leading to IGHD type 1b in a 13-months old Greek girl, the youngest patient with a GHRH-R mutation reported so far.

## Case Report

A 13-month old girl was admitted to our department due to failure to thrive. She was the second child of healthy, unrelated parents, whose heights were 190 cm (father) and 175 cm (mother). An ethical review board approval and informed consent from both parents of the proband presented here were obtained, in accordance with national laws.

**Figure 1:**
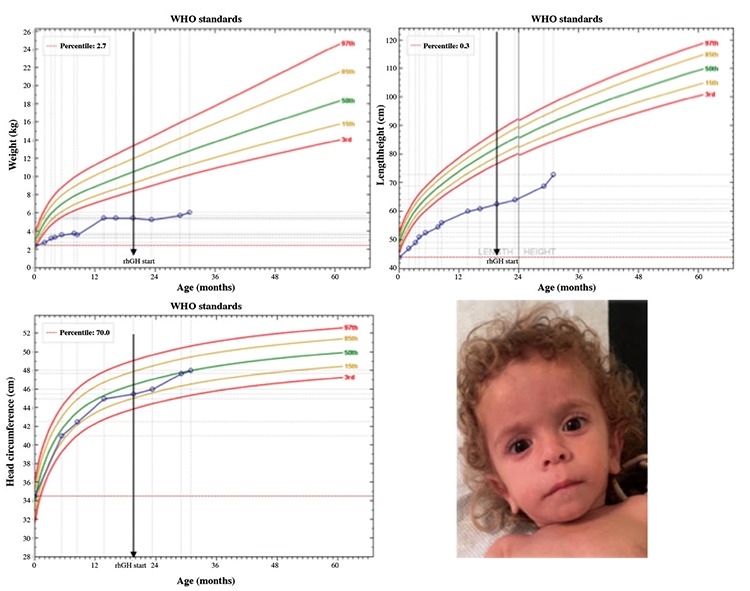
Growth chart for weight-for-age, height-for-age and head circumference-for-age (Anthro World Health Organization software) along with a clinical photograph of the patient at 15 months of age showing typical facial features of a patient with growth hormone deficiency WHO: World Health Organization

The patient was the product of a 37 weeks gestation. During the 4^th^-8^th^ gestational week, the mother experienced vaginal bleeding. Intrauterine growth retardation was diagnosed in the 8^th^ gestational week due to placental insufficiency. Additionally, the mother admitted she was smoking during the entire pregnancy period. The newborn was asymmetrical and small for gestational age (SGA), with a birth weight of 2420 g (<3^rd^ percentile, z-score: -1.93), and a length of 44 cm (<3^rd^ percentile, z-score: -2.76) (Figure 1). Head circumference was 34.5 cm (70^th^ percentile, z-score: 0.52). She was partially breast-fed during the first 30 days of life. Due to the infant’s unwillingness to take formula milk, she was transferred to the pediatric gastroenterology department where a 24-hour nasogastric tube was placed at the age of nine months and hypercaloric oral supplements were administered, without significant effect on body weight gain (Figure 1).

On physical examination, at 13 months of age, the infant was small and skinny, not resembling obese GH deficient neonates. Her length was 60 cm (<3^rd^ percentile, z-score: -6.03) and her weight 5470 g (<3^rd^ percentile, z-score: -4.35). Head circumference was 45 cm (40^th^ percentile, z-score: -0.27) and head shape was triangular with open fontanelles. Hair was very sparse and ears were low set. Nasal bridge was hypoplastic and dental development was significantly retarded (one tooth). Motor milestones were delayed; she was able to sit but could not stand. Systematic clinical examination of the heart, lungs and abdomen did not reveal abnormal findings.

Complete blood count, haemoglobin levels and glucose concentrations as well as renal function were within the normal range for her age. Karyotype analysis showed a normal female genotype of 46 XX. Thyroid and adrenal hormone levels were normal. Serological indices for celiac disease or food allergy were negative. Serum GH response to clonidine, glucagon and arginine stimulation tests revealed very poor response, with a peak GH value of 4.77 ng/mL, demonstrating IGHD (Table 1). An IGF-1 generation test after administering GH at a dose of 33 µg/kg for four consecutive days showed low IGF-1 levels with a modest response (Table 1). After 12 months of GH treatment, serum IGF-1 level rose to 23 ng/mL. Bone age was two months at the chronological age of 13 months. Magnetic resonance imaging of the brain revealed a normal pituitary gland and normal hypothalamus.

At the chronological age of 19 months the patient was administered GH at a starting dose of 0.28 mg/kg/week, subcutaneously. After ten months, GH dose was increased to 0.35 mg/kg/week. At the age of 22 months she started to walk. At the chronological age of 24 months she presented a 12 month phalangeal and a nine month carpal bone age. After ten months of medication she gained 7 cm in length (8.14 cm/year), 300 g in weight and her head circumference had increased by 2.2 cm. After one year of treatment (at chronological age of 31 months) the patient had achieved a length of 73.5 cm (<3^rd^ percentile, z-score: -5.2), a weight of 6100 g (<3^rd^ percentile, z-score: -5.65) and a head circumference of 48 cms (50^th^ percentile, z-score: -0.02) (Figure 1).

Due to the facial features of the patient, Silver-Russell syndrome has been suspected. The absence of the clinical criteria of Price et al (7) along with a deletion/duplication analysis with array genomic hybridization, Silver-Russell syndrome was excluded. Additionally, presence of intrauterine growth retardation, along with facial characteristics and delayed eruption of teeth, could suggest a possible diagnosis of 3M syndrome. Triple whole exome sequencing (WES) of the affected girl and parents (CentoXome GOLD^®^) using Illumina technology was performed. No mutation on *CUL7*, *OBSL1* or *CCDC8* genes, the mutations leading to 3M syndrome were found. A novel homozygous nonsense variant in the *GHRH-R* gene, the c.97C>T (p.Gln33*) was detected. The observed variant creates a premature stop codon and is classified as likely pathogenic-class 2 variant. Parental genotyping detected the novel variant in the mother in a heterozygous form, but it was not found in the father. It is suspected that a large deletion not detectable by WES in the paternal allele is present. The detected c.97C>T variant of the *GHRH-R* gene has never been reported before and not listed, in the CentoMD.

## Discussion

The present report describes an unknown GHRH-R mutation, in an infant girl of Greek origin, with a clinical appearance resembling a SGA state, rather than congenital GHD (8,9). To the best of our knowledge this is the youngest patient described in the literature to date with a mutation of the *GHRH-R* gene. The patient presented with a skinny appearance and showed a low IGF-1 response to an IGF-1 generation test. These two unexpected findings probably relate to caloric insufficiency caused by placental insufficiency (10), possibly due to the mother smoking throughout the pregnancy.

Some of the causes of congenital IGHD are *GHRH-R* gene defects. These gene defects are being described more frequently in the literature (11). Currently more than thirty-three mutations in the *GHRH-R* gene have been shown to cause impaired GHRH-GH-IGF-1 axis function, whereas no mutations in the *GHRH* gene have been reported. The large majority of these cases showed an autosomal recessive model of inheritance (12). Mutations of GHRH-R, classified into six different types, cause defective GHRH functionality (13). Null-type GHRH-R mutations lead to unmeasurable IGF-1 levels and are accompanied by mild ocular disorders (14). Missense GHRH-R variants -such as p.G369V or p.T257A- result in partial loss of receptor function due to defective ligand binding and milder phenotypes, occasionally accompanied by hypoglycemia (15). Splice site mutations of untranslated and coding regions have been reported to lead to gross indels with loss of 5’ regulatory/exon 1 region, leading to fully impaired GHRH-R expression (12). Other splice-disrupting, single nucleotide polymorphisms like intronic mutations, lead to instability of the produced mRNA, truncated GHRH-R and autosomal recessively inherited IGHD (16). Nonsense type mutations lead to loss-of-function changes (17), whereas functional variants of the GHRH-R promoter affect promoter activity and thus decrease expression of the receptor gene (18).

Herein, we present a previously undescribed homozygous *GHRH-R* gene mutation, c.97C>T (p.Gln33*) in a child with IGHD type 1b. Clinical and biochemical phenotype of the affected individual comprises severe short stature, low weight gain, low maximum GH values after a battery of provocation tests, inadequate response to IGF-1 generation testing, normal brain imaging and growth acceleration after GH therapy. The reported mutation is predicted to lead to a premature stop codon and thus it signals the termination of translation of the relevant messenger RNA. Defective translation of the gene results in a shorter encoded protein and thus an impaired form of GHRH receptor. The novel mutation probably affects the receptor in terms of both sequence and structure, leading to the inhibition of GHRH binding to its receptor and thus to disruption in GH secretion signaling. According to the recommendations of the American College of Medical Genetics and Genomics, the novel mutant is classified as likely pathogenic, class 2.

Since the variant was detected in the maternal DNA in heterozygous state, but not in the paternal genome, the precise pattern of inheritance can not be confirmed. A suggested large deletion in the exact region of the paternal *GHRH-R* locus could explain the inability to detect the mutation via father WES analysis. Nevertheless, based on the finding of a healthy, unaffected heterogyzous mother, it could be assumed that the variant presented here, p.Gln33*, represents an autosomal recessive inheritance trait.

Intrauterine growth restriction is closely associated with placental quality, functionality and therefore adequacy. Multiple layers of associations have been suggested for the causes of fetal growth restriction and SGA offspring. *In utero* exposure to tobacco constitutes a known risk factors for both conditions. From a meta-analytic approach, even exposure to tobacco smoke during pregnancy is associated with low birth weight (19). Exposure of offspring to tobacco metabolites through maternal milk during infancy has also been suggested (20). Nevertheless, cohort studies have provided evidence that maternal smoking during pregnancy or early infantile life exert a long-term negative effect on growth (21). The presented case constitutes a paradigm of mixture between nature and nurture. Apart from the detected defect in *GHRH-R* gene sequence, *in utero* environment and after birth conditions have contributed to the phenotype. Synergistic effects of genetics and epigenetic conditions are not fully understoood and remain to be elucidated.

In conclusion, we report a novel homozygous c.97C>T (p.Gln33*) GHRH-R mutation determined in a Greek infant girl with IGHD. Heterozygosity of the reported variant was not associated with pathological phenotypes in the unaffected family member c.97C>T.

## Figures and Tables

**Table 1:**
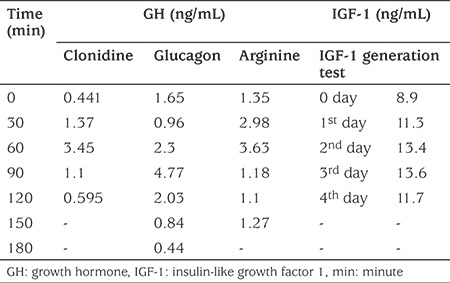
Growth hormone provocation and insulin-like growth factor 1 generation test values
